# A Dynamic Energy Budget (DEB) based modeling framework to describe tumor-in-host growth inhibition and cachexia onset during anticancer treatment in *in vivo* xenograft studies

**DOI:** 10.18632/oncotarget.27960

**Published:** 2021-07-06

**Authors:** Elena Maria Tosca, Maurizio Rocchetti, Paolo Magni

**Affiliations:** ^1^Department of Electrical, Computer and Biomedical Engineering, University of Pavia, Pavia I-27100, Italy; ^2^Consultant, Milano, Italy

**Keywords:** PK-PD modeling, tumor-growth-inhibition model, dynamic energy budget theory, cancer cachexia, xenograft model

## Abstract

Cancer anorexia-cachexia syndrome (CACS) is a very severe complication of cancer for which an adequate therapeutic strategy has not yet been defined. Recently, a notable number of new animal models of human CACS has been made available for translational purposes. Under the assumption that tumor-induced adaptations of host metabolism and tumor-host energetic competition play a major role in CACS (together with possible toxicities induced by the anticancer treatment), we developed a new Dynamic Energy Budget (DEB)-based framework, modeling tumor-in-host growth dynamics and cachexia onset in preclinical animal models during anticancer treatments. The tumor-in-host modeling approach was successfully applied on a multitude of *in vivo* preclinical studies involving different host species, tumor cell lines, type of anticancer agents and experimental settings among which standard xenograft studies. Obtained results strongly suggested the adoption of the tumor-in-host DEB-based approach in the preclinical oncological setting for a joint assessment of drug efficacy and toxicity and for a better design of the experiments. Further applications of the DEB-based approach to the context of poly-targeted combination therapy, anti-cachectic treatments and preclinical to clinical translation are under investigation with extremely encouraging preliminary results.

## INTRODUCTION

Cancer anorexia-cachexia syndrome (CACS) is a very severe complication of cancer that affects the majority of cancer patients and it is responsible of 20% of their death [1]. It is defined as a multifactorial syndrome characterized by an ongoing loss of skeletal muscle mass (sarcopenia) with or without loss of fat mass that cannot be fully reversed by conventional nutritional support and leads to progressive functional impairment [2]. The pathophysiology of cachexia is characterized by a negative energy and protein balance that is driven by a combination of starvation (anorexia) and abnormal metabolism, seemingly induced by multiple factors related to both tumor progression and drug toxic effects [1, 3]. The impact of CACS on the quality of life of cancer patients is devastating. In addition, relevant body weight loss (BWL) is generally associated to greater side effects, poor response to treatment and consequently early death.

Even if a number of clinical trials in cancer cachexia have been conducted, none of them resulted in a regulatory approval of a therapeutic strategy [4]. The design of an effective treatment has been limited by both the difficulties in recruiting patients for clinical studies and the insufficient knowledge of the CACS pathogenesis [5]. This opened a debate about the availability of appropriate in vivo preclinical models [6] and in the recent years a notable number of new animal models of human CACS that may be useful to circumvent the clinical limitations, expand understanding of underlying pathogenic mechanisms and explore the effectiveness of prospective treatments for translational purposes, have been developed [7–9].

For these reasons and starting from the assumption that host metabolic adaptations induced by tumor progression and tumor-host energetic competition play a major role in the CACS development (together with possible toxicities induced by anticancer treatments), we developed a modeling approach based on the Dynamic Energy Budget (DEB) theory [10]. It is a formal metabolic theory that, using simple and straightforward rules concerning the uptake, storage and utilization of energy, is able to describe in a unique quantitative framework all the living organisms, from single cells to animals and humans. A first attempt to a tumor-in-host growth model based on the DEB theory was proposed by Van Leeuwen [11, 12]. This modeling approach was developed on the hypothesis that host features, such as cell proliferation rates, caloric intake, metabolism and energetic conditions, significantly influence tumor growth [13] and that, in turn, tumor progression has relevant implications for host physiology. Even if the van Leeuwen model accounted for the reciprocal dependence of tumor growth and host physiology, it was too complex to be applied in the preclinical settings where routinely simple experiments are conducted. In addition the effects of anticancer treatment on both body host and tumor mass were not taken in account.

This editorial aims to summarize the research that in the last 7–8 years has led to the development of a new DEB-based framework modeling the tumor-in-host growth dynamics and the cachexia onset in preclinical animal models during anticancer treatments [14–16]. The model was successfully tested on a multitude of *in vivo* preclinical studies involving different host species (mice and rats), tumor cell lines, type of anticancer agents (cytotoxic and cytostatic) and experimental settings between which standard xenograft studies typically performed to assess the anticancer efficacy of investigated compounds [17, 18].

### The Tumor-in-Host DEB-based modeling framework

**Figure 1:**
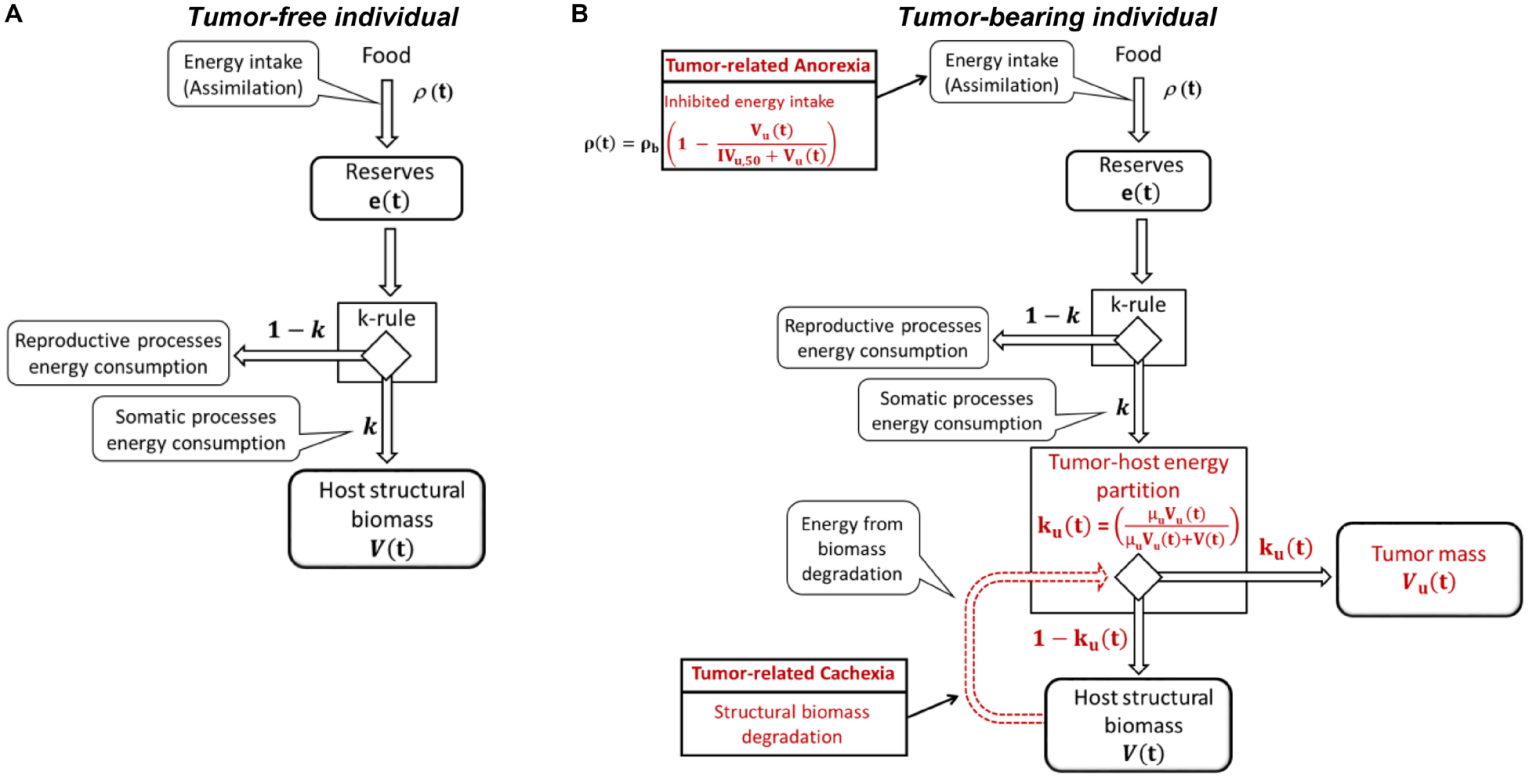
Energy fluxes in tumor-free (**A**) and tumor-bearing (**B**) individual.

Following the van Leeuwen work [11, 12], the approach here discussed adopts the DEB theory as general framework to describe the host organism. Host is modeled by its body weight that is composed by the structural biomass (approximable with skeletal muscle) and the energy reserve (approximable with adipose tissue). The dynamics of these two components simply follow from an energy balance between the main physiological processes such as assimilation, energetic consumption, somatic and reproductive processes (Figure 1A). More in details, energy assimilation is considered proportional to the surface area of the structural biomass through the food-supply coefficient, *ρ(t)*. Assimilated energy is stored in the reserve, *e(t)*, from which it can be made available for physiological processes. Once mobilized, the energy is split into two branches: a fixed fraction, *k*, is allocated to somatic processes (maintenance and growth of structural biomass), while the remaining fraction, 1-*k*, is available for reproductive processes (*k*-rule). Maintenance energetic costs, *m*, are proportional to structural volume, *V(t)*, and have the priority on the growth, *g*, which energetic costs are proportional to the instant variation of structural biomass.

In this framework, tumor is conceived as an additional energy demanding component, able to subtract a fraction, *k_u_(t)*, of the energy available for the somatic processes. It uses this energy for its maintenance, *m_u_*, and growth, *g_u_* (Figure 1B). The tumor-host energy partition fraction depends on tumor volume, *V_u_(t)*, on structural biomass volume and on a coefficient of “tumor gluttony”, *μ_u_*, that quantifies the efficiency of tumor cells in extracting energy in comparison of normal cells. As the tumor exploits host resources, in certain conditions, the organism has to reduce its growth rate and even degrade its structural biomass to survive and, at the same time, to satisfy the tumor energy demand (tumor-related cachexia). The degradation rate increases until a maximum threshold is reached. This condition can be further worsened by the negative impact of tumor progression on host assimilation (tumor-related anorexia).

**Figure 2:**
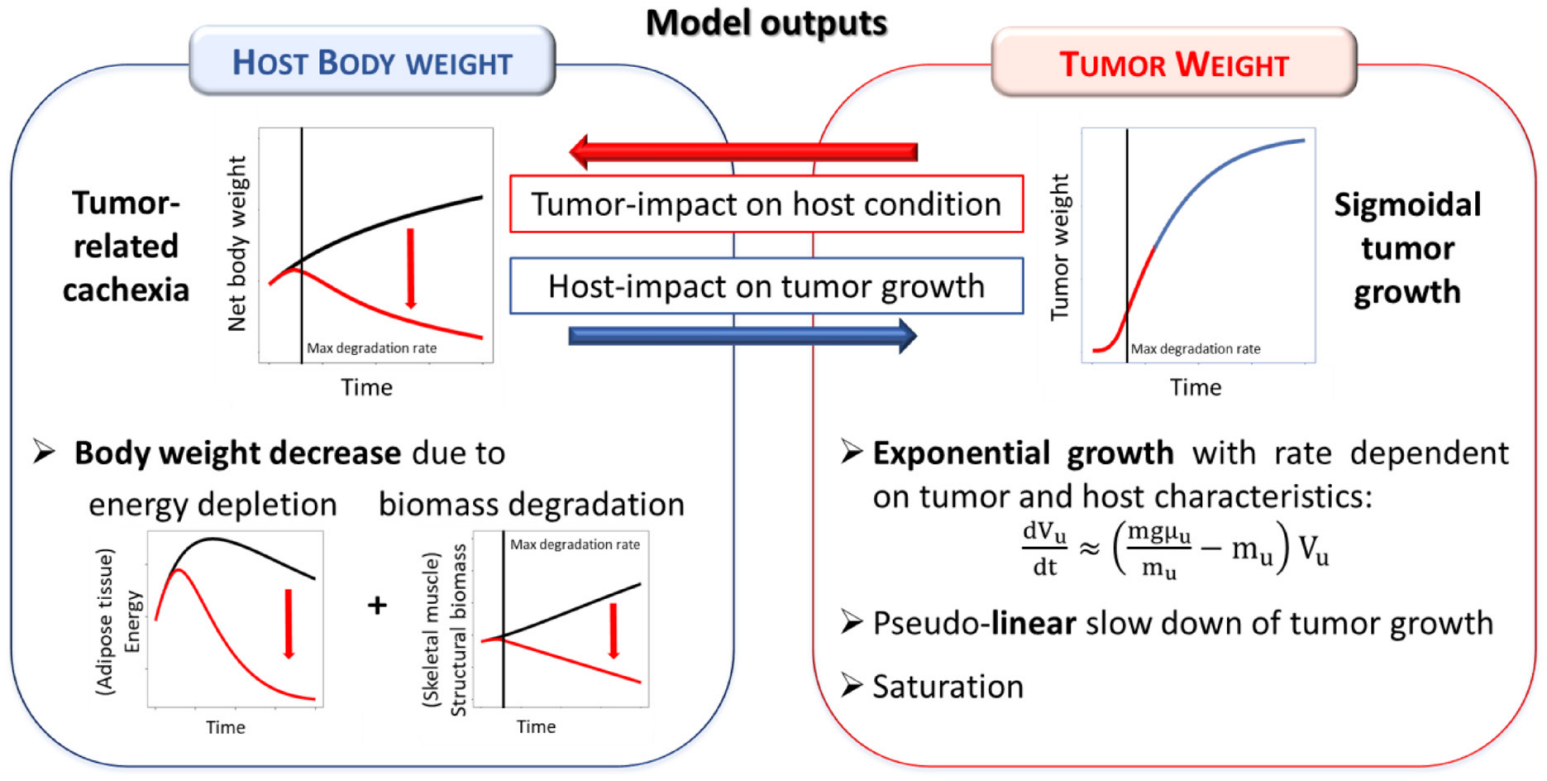
Summary of the tumor-in-host interactions as modeled in the tumor-in-host DEB-based framework.

The two observable quantities predicted by the model (model outputs) are the net body weight of the host organism (i.e., the weight of the host excluding the tumor mass) and the tumor weight. Tumor and host weight dynamics are not independent on each other, but they are characterized by a mutual influence (Figure 2). In particular, tumor growth induces host body weight decreases that are due to both energy depletion (mainly reduction of adipose tissue) and degradation of structural biomass (mainly wasting of skeletal mass). On the opposite, these host energetic changes influence tumor growth that, consequently, follows a sigmoidal behavior [16]: initially, tumor grows exponentially with a rate dependent on both tumor and host characteristics $\left(\tilde{{\text{λ}}_{0}}\right)$; then, there is a slowdown of the growth rate eventually followed by a saturation due to the depletion of the host energy resources. The most interesting aspect is that the dynamics of tumor growth are not imposed by empirical assumptions but simply follow from energetic interactions between tumor and host.

This tumor-in-host DEB-based model demonstrated to be an optimal tool to jointly describe the tumor and host body weight dynamics observed in preclinical xenograft studies, typically performed to assess the anticancer efficacy of investigated compounds. In these studies, in addition to tumor weight, also the body weight of host organism (typically mice or rats) is usually monitored and significant BWL are frequently observed and used as stopping rule. In addition to the description of the tumor-in-host dynamics in absence of treatment, the DEB-based model provides a general framework that has been exploited, adapted and extended to account for different kinds of anticancer compound characterized by different mode of action. In all the cases, plasma concentration of the considered drug, simulated by a specific PK model, drives the effects of the anticancer treatment.

### Tumor-in-Host DEB-TGI model for cytotoxic agents

For chemotherapeutic agents, the model supposes that drug exerts two effects. First drug cytotoxicity is modeled by a direct killing effect on tumor cells: a fraction of tumor cells hidden by the drug becomes not-proliferating and heads towards death through several damage stages [19]. As in the Simeoni TGI model [20, 21], cytotoxic effect is proportional to drug concentration through a parameter K_2_, that represents the drug antitumor potency. The second effect is an inhibitory effect (inhibitory Imax model) on host assimilation. It accounts for temporary side effects of the drug treatment, such as weaking, loss of appetite or limited assimilation (drug-related anorexia), that can lead to an increased BWL (drug-related cachexia). So, the half maximal inhibitory concentration, IC50, represents a quantitative measurement of drug toxicity on host body.

**Figure 3:**
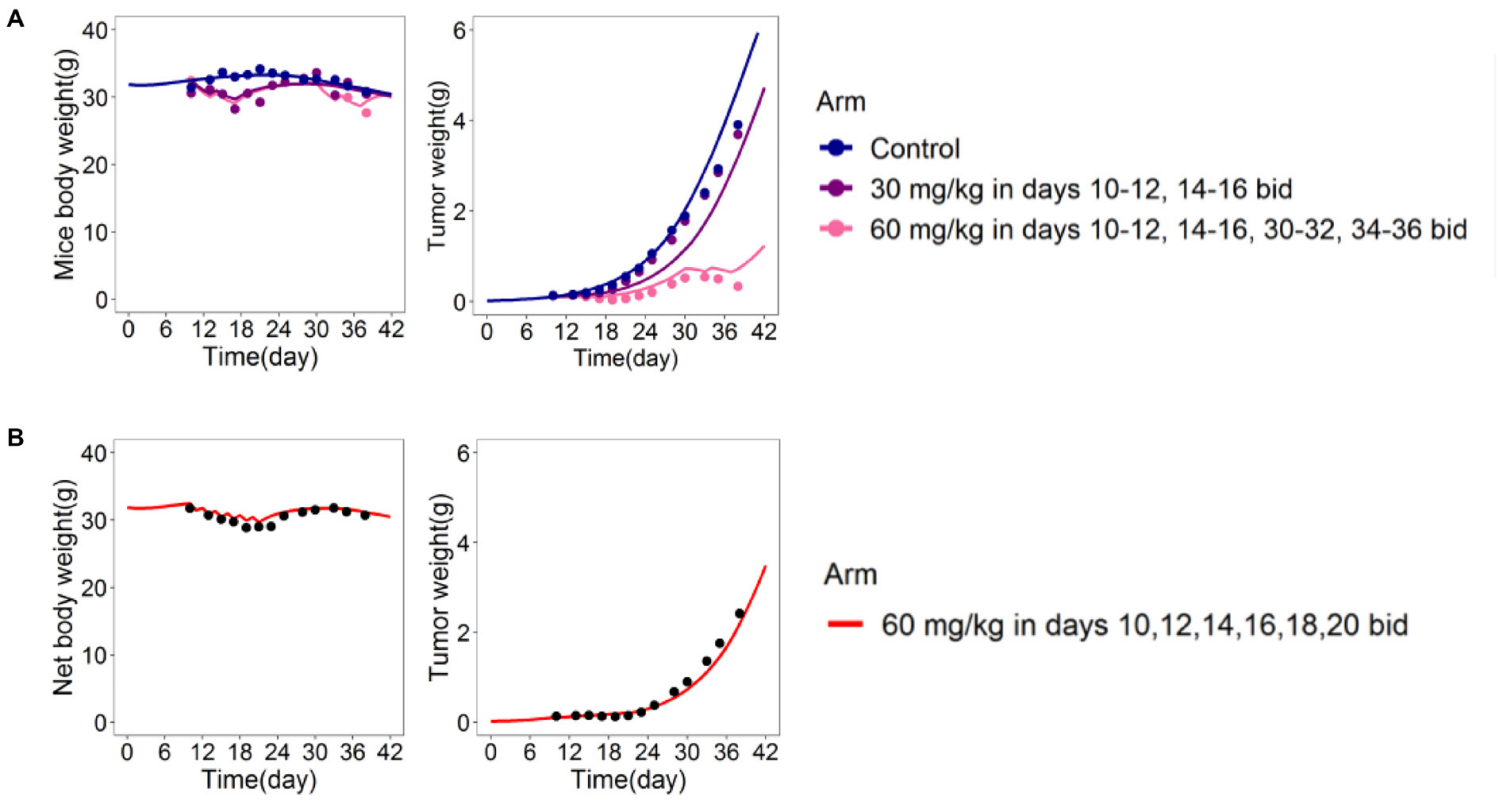
DEB-based tumor-in-host model predictive power assessed in xenograft mice [16]. Model was fitted on mice body and tumor weight data from control and 2 treated arms (**A**) and then used to predict host and tumor responses to a different administration protocol (**B**).

The tumor-in-host DEB-TGI model for cytotoxic agents was used to analyze two sets of experimental data. The first is related to eight xenograft studies involving three tumor cell lines performed in mice for the assessment of drug efficacy [16]. Results demonstrated the model ability to simultaneous describe tumor and host body weight growth both in control (untreated animals) and treated mice with new anticancer candidates and well-known drugs (paclitaxel, 5-FU, cisplatin, vincristine, vinblastine and gemcitabine) administered at different dose and schedules (Figure 3A). In addition, the capability of the DEB-based model to predict tumor and host body response to new administration protocols was proven. In fact, the model, previously identified on data from control and two treated arms (Figure 3A), was used to predict a new arm involving the same compound administered by a different dose and schedule (Figure 3B).

**Figure 4:**
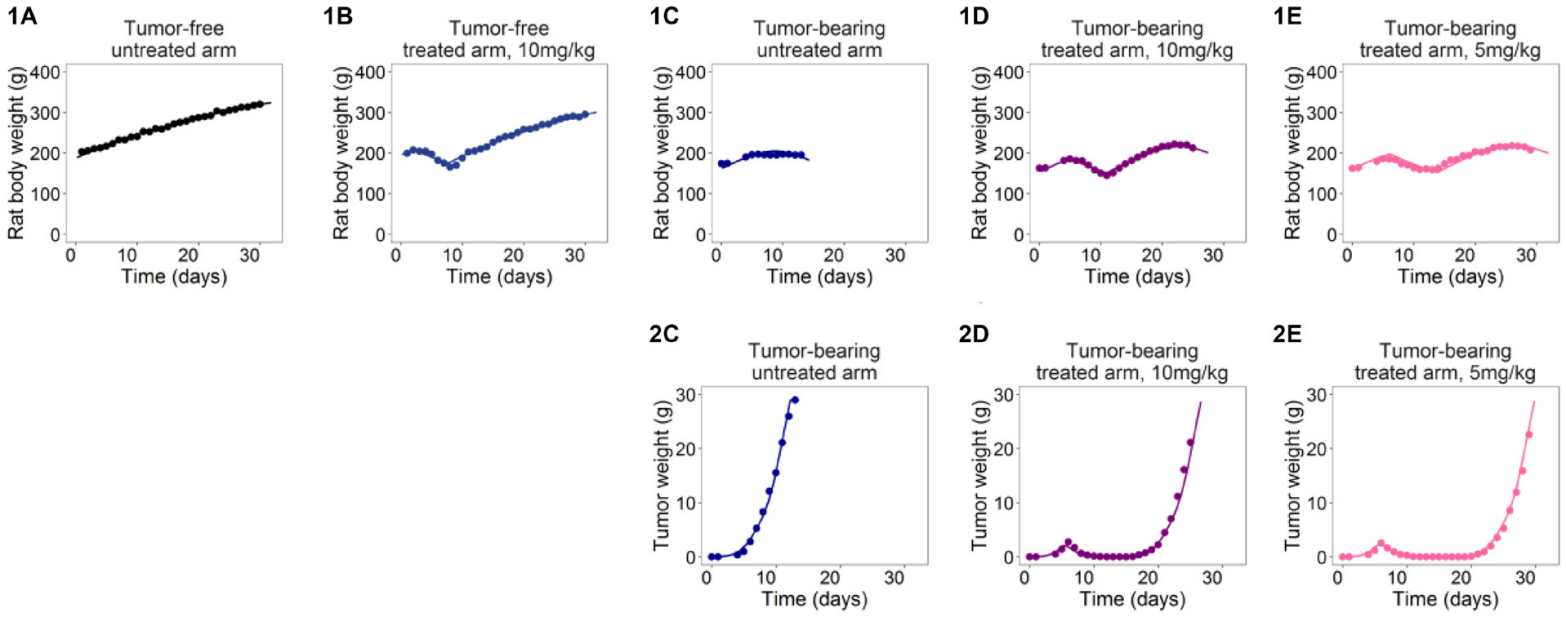
Plots with average measures (dots) and typical model predictions (lines) for host body (**1A**–**1E**) and tumor weight (**2C**–**2E**) curves in the five arms of Etoposide studies on Wistar Rats [15].

The second set of data regards an *in vivo* experiment performed to assess the Etoposide activity on Wistar rats [15]. The study was composed by five arms: the standard control group (tumor-bearing untreated animals), two tumor-bearing groups treated with Etoposide administered with two different protocols and two additional arms composed by treated and untreated tumor-free animals. A slightly revised model formulation, combined with a population non-linear mixed effect approach and with the use of intratumoral concentration as driver of tumor kinetics, successfully described the Etoposide effects on Wistar rats (Figure 4). The well-design experiment, including treated and untreated tumor-free animals, allowed to fully exploit model capabilities to describe and discern all the *in vivo* tumor-in-host growth dynamics including both tumor- and drug-related anorexia and cachexia. This unusual and reach experimental design played an important role in discerning all the contributions on the tumor and host dynamics summarized in Figures 1 and 2. Indeed, the model was able to describe the uncontrolled tumor growth in control animals (Panel 2C) and its schedule-dependent inhibition due to the Etoposide effect (Panels 2D and 2E). For what concerns the host, the model accounted for its body growth in absence of tumor mass (untreated tumor-free animals, Panel 1A) and its slowdown due to the impact of tumor-related cachexia (untreated tumor-bearing animals, Panel 1C). This was discerned from the BWL due to the drug-related anorexia that was highlighted in the treated tumor-free group (Panel 1B). Finally, the contribution of both the effects of tumor-related cachexia and drug-related anorexia were also well described in the tumor-bearing treated groups (Panels 1C and 1D).

### Tumor-in-Host DEB-TGI model for cytostatic agents

Cytostatic agents, such as anti-angiogenic compounds, modulate tumor growth without exert direct cytotoxicity on cancer cells. Thus, independently on the drug specific mechanism of action, cytostatic effect can be modeled as a reduction of tumor ability to survive and proliferate. In particular, for anti-angiogenic compounds it is assumed that the decreased tumor vascularization leads to an inhibition of tumor energy supply, modeled as an inhibitory function (inhibitory Imax model) on the fraction *K_u_(t)* [14].

**Figure 5:**
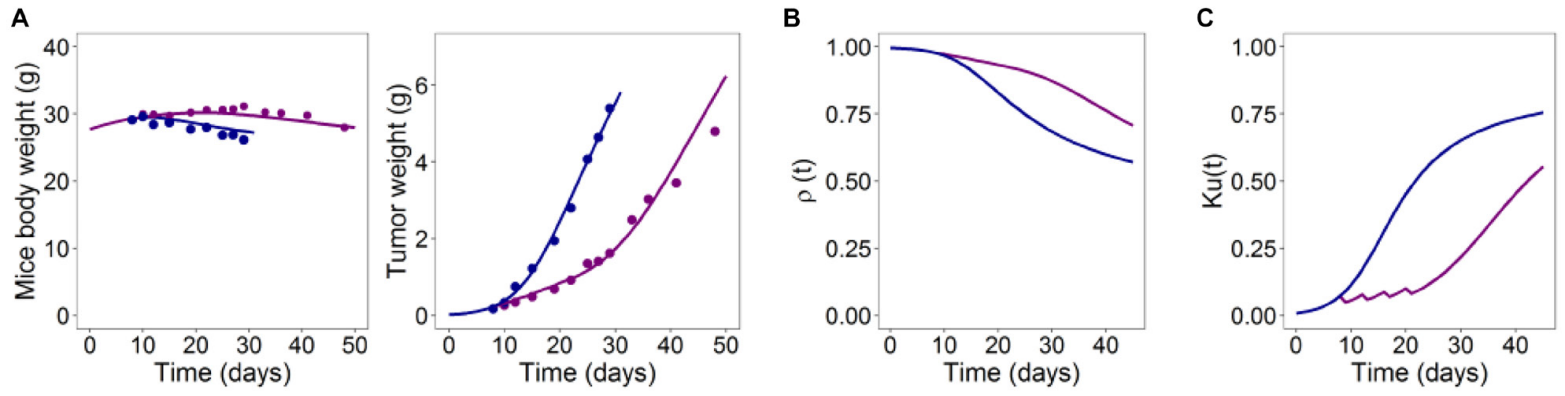
(**A**) Typical model predictions (lines) for mice body and tumor weight curves together with average observed data (dots) for control (blue) and treated arms (purple). (**B**) Time course of host assimilation. (**C**) Time course of the energy fraction *k_u_(t)* [14].

The tumor-in-host DEB-TGI model for anti-angiogenic agents together with a population approach was successfully applied to seven xenograft mice studies assessing the effect of Bevacizumab (Avastin) on three different tumor cell lines [14]. Because Bevacizumab does not exert any toxic effect in mice, in this case host assimilation is affected only by tumor progression (tumor-related anorexia). However, drug treatment acts on the energy repartition between tumor and host and, thus, exerts an indirect anti-cachectic effect on the host. The model was able to describe the modulation of tumor growth in treated groups in comparison to control animals as well as the positive anti-cachectic effect of anti-angiogenic treatment on the host body weight (see panels A in Figure 5 for an illustrative example). In addition to an excellent description of the data, the model provides quantitative measurements about several outcomes of interest such as the severity of tumor-related anorexia, the tumor-host energy distribution or the drug anticancer potency. In the analyzed studies, the impact of tumor progression on host body weight was quite relevant, with an observed BWL up to 14% in the control animals. The mechanistic DEB-based model allowed to determine the time course of the two components that contribute to the overall reduction of the energy available for host physiological processes, i.e., the inhibition of host energy intake due to tumor-related anorexia (Panel B) and the tumor-host energy partition coefficient (Panel C).

Finally, the efficacy metrics quantifying the drug anticancer potency allowed not only to compare the antitumor effect of Bevacizumab in the different tumor cell lines but also to recognize a decreased Bevacizumab efficacy during prolonged treatments. Supported by literature data, a hypoxia triggered resistance model was developed allowing to describe tumor and host response to administration protocols of different durations. In particular, the integration of the resistance mechanism enabled the model to grasp the decreasing efficacy of Bevacizumab therapy and to correctly predict the length of the initial response phase. Obtained results suggested the use of the model to evaluate and compare different administration protocols including continuous or intermittent schedules.

### Peculiarities and new horizons of the tumor-in-host DEB-based modeling framework

Finding the best compromise between efficacy and toxicity is the goal of any anticancer therapy. In absence of appropriate models that consider both the tumor and host body interactions (tumor-in-host models) and the effects of an anticancer drug therapy, this efficacy/toxicity evaluation is based only on heavy and time-consuming trial-and-error procedures. The tumor-in-host DEB-based modeling framework, that we recently developed and tested in different settings, including the estimate of the possible effects on CACS of anticancer treatments, provides a new tool to cope up with this well-acknowledged gap.

The approach was able to simultaneously model tumor and host organism interactions during anticancer treatments integrating all the different aspects characterizing the *in vivo* TGI studies: drug cytotoxic or cytostatic activity on tumor, onset of drug- and tumor-related cachexia and anorexia and influence of host condition on tumor growth. It allowed for the first time to investigate separately BWL due to the tumor progression and to treatments, providing in this way, an unbiased estimate of the anticancer drug efficacy that results to be disentangled from the possible effect on tumor growth due to of host energy depletion. The strength of this approach was further consolidated by its broad applicability. Indeed, it was successfully applied to different host species, tumor cell lines and anticancer agents based on different mechanisms of action. Everything using experimental data commonly generated during an anticancer drug development process. These findings strongly suggested the adoption of the tumor-in-host DEB-based approach in the preclinical oncological setting for a joint assessment of drug efficacy and toxicity on animal BW and for a better design of the experiments.

Finally, new investigations about further applications of our tumor-in-host DEB-based approach are running and preliminary results are more than encouraging. For example, the applicability of the DEB-based approach could be further extent to the context of poly-targeted combination therapy [22–24]. The modeling framework would be suitable enough to describe the effect of different molecular target-agents which combinations are currently under careful attention to overcome the issue of resistance development. An example is the co-administration of c-MET inhibitors and EGFR-tyrosine kinase inhibitors for the treatment of non-small-cell lung cancer [25]. In this context, the tumor-in-host nature of DEB approach would allow to evaluate and predict possible drug-drug interactions (synergistic, antagonistic or additive behavior) based on both tumor and host body weight dynamics.

The growing interest in the CACS, for which there is lack of successful treatments, suggests testing the use of the DEB-based framework as tool to analyze data generated during preclinical studies on animal models of cancer anorexia and cachexia. Preclinical experiments performed on rodents and involving different tumor models are available in the literature [6]. In addition to tumor and host body weights, data include daily or cumulative food intake, weights/diameter of gastrocnemius muscle and of epididymal adipose tissue (two measurements representative of the animal body composition) for tumor-bearing and tumor-free animals. The tumor-in-host DEB-based model, adequately identified only on tumor and host body weight, would be able to describe and predict the key endpoints of cancer-cachexia in animal studies such as tumor burden, BWL, food-intake reduction and body composition changes induced by tumor progression.

Lastly, specific modeling efforts are focusing on taking advantages of the DEB-based paradigm to develop a preclinical to clinical translational approach. Indeed, despite its complexity, during the measurable phase human tumor growth displays an S-shaped growth pattern characterized by an exponential growth rate, λ [26, 27] and an upper tumor burden limit (~10^12^–10^13^ cells ≈ 1–10 kg) in which the tumor become lethal for the host. In absence of biological or molecular predictive markers, tumor volume doubling time (TVDT ≈ ln(2)/λ) is considered a non-invasive assay extremely useful for important strategies such as screening programs, survival data analysis during clinical trials or estimation of risk period of late recurrences. A scaling strategy that allows to translate the DEB-based S-shape tumor-in-host growth from xenograft mice to human and to predict human TVDT is currently under evaluation. The idea is to perform the translation in several steps that exploit the nested structure of the DEB-based tumor-in-host model. First, the host growth would be scaled in absence of tumor adopting scaling rules based on body size and life-span and using only weight growth curves of tumor-free individuals. Secondly, the scaling rules previously identified would be applied to scale from animal to human the tumor-in-host growth. Following this strategy, model estimates obtained on xenograft studies would be used to predict TVDTs observed in patients affecting by several cancer types (melanoma, ovarian, breast, gastric, colon, pancreatic and lung cancer). Based on some preliminary results, it seems that there is a good agreement between model predicted and literature clinical TVDTs with an absolute average fold error always < 1 and an *r*^2^ > 0.8. Moreover, the scaled model would be used to qualitative predict human tumor-in-host kinetics: model would predict body weight reductions in line with cachectic state often observed in cancer patients [1–3] and, in particular, a deep BWL in correspondence of a tumor volume generally considered as a lethal burden.
